# Micro-CT and histological investigation of the spatial pattern of feto-placental vascular density

**DOI:** 10.1016/j.placenta.2019.09.014

**Published:** 2019-12

**Authors:** R. Aughwane, C. Schaaf, J.C. Hutchinson, A. Virasami, M.A. Zuluaga, N. Sebire, O.J. Arthurs, T. Vercauteren, S. Ourselin, A. Melbourne, A.L. David

**Affiliations:** a Dept. Med. Phys. Biomed. Eng., University College London, UK; b Institute for Women's Health, University College London, UK; c NIHR UCL GOS Institute of Child Health Biomedical Research Centre, University College London, UK; d Department of Histopathology, Great Ormond Street Hospital for Children, NHS Trust, London, UK; e Paediatric Radiology, Great Ormond Street Hospital for Children NHS Foundation Trust, London, UK; f School of Biomedical Engineering & Imaging Sciences, King's College London, UK; g Clinical Investigation Centre of Nancy, France; h Data Science Department, EURECOM, Biot, France

## Abstract

**Introduction:**

There are considerable variations in villous morphology within a normal placenta. However, whether there is a reproducible spatial pattern of variation in villous vascular density is not known. Micro-CT provides three-dimensional volume imaging with spatial resolution down to the micrometre scale. In this study, we applied Micro-CT and histological analysis to investigate the degree of heterogeneity of vascularisation within the placenta.

**Method:**

Ten term placentas were collected at elective caesarean section, perfused with contrast agent and imaged whole with Micro-CT. Eight full depth tissue blocks were then taken from each placenta and imaged. Sections were taken for histological analysis. Data was analysed to investigate vascular fill, and vascular density in relation to location from cord insertion to placental edge at each scale.

**Results:**

Whole placental imaging revealed no spatially consistent difference in villous vessel density within the main placental tissue, although there was a great degree of heterogeneity. Both block imaging and histological analysis found a large degree of heterogeneity of vascular density within placentas, but no strong correlation between villous vascular density and block location (r_s_ = 0.066, p = 0.7 block imaging, r_s_ = 0.06, p = 0.6 histological analysis).

**Discussion:**

This work presents a novel method for imaging the human placenta vascular tree using multiscale Micro-CT imaging. It demonstrates that there is a large degree of variation in vascular density throughout normal term human placentas. The three-dimensional data created by this technique could be used, with more advanced computer analysis, to further investigate the structure of the vascular tree.

## Highlights

• Micro-CT and histological investigation of vascular density in the placenta.
• There is a large degree of variation in vascular density throughout placentas.
• This imaging has potential for future spatial investigation of the 3D vascular tree.

## 1 Introduction

Fetal blood arrives at the placenta via two umbilical arteries, is transported across the placental surface via chorionic arteries, then passes deep into the placenta via stem arteries. From these dense vascular trees arise forming complex, multi-branching vascular beds [1], bringing fetal blood in close proximity with maternal blood, allowing exchange [2]. Important obstetric pathologies, including pre-eclampsia and fetal growth restriction, are associated with changes in the villous vascularisation of the placenta [[3], [4], [5], [6]]. Improving our understanding of normal placental vascularisation and the changes seen in pathology may improve our understanding of these diseases, and our ability to diagnose and treat them.

There are considerable variations in villous morphology within a normal placenta [7]. However, whether there is a relationship between variation in villous vascular density and tissue location within the placenta in regard to umbilical cord insertion and placental edge is unclear. Histological analysis by Fox et al. investigating the number of hypovascular or avascular villi, their measure of feto-placental vascularisation, in relation to tissue location within the placenta and found no statistically significant relationship in normal placentas [7]. However, they did show an increasing number with distance from cord insertion (156 centrally vs 222 peripherally) [7], suggesting there may be reduced vascular density in the placental periphery. Mayhew et al. [8] did not reproduce this, finding no difference in villous vascular density with tissue location in relation to cord insertion and placental edge.

Micro-Computed Tomography (Micro-CT) provides three-dimensional volume imaging with spatial resolution down to the micrometre scale, although magnification is at the cost of field of view. It has the advantage of being non-destructive allowing further tissue analysis with other imaging or histological techniques. Micro-CT has already been shown to be effective in investigating the fetoplacental circulation of mouse placentas, demonstrating the growing complexity of the vascular tree with increasing gestational age [9], and the effect of polycyclic aromatic hydrocarbons on the branching structure and tortuosity of the tree [10]. In human placenta, the technique has been used to measure placental vascular density in small blocks of tissue [11], and demonstrate reduced vascular density in fetal growth restriction compared to normally grown controls [12]. Imaging of the whole human placenta, using a corrosion technique, has also been investigated [13], finding a significantly smaller number of chorionic artery branches [13] and longer venous and shorted arterial vasculature in fetal growth restriction compared to normal placentas [14]. Standard Computed Tomography angiography has been used to investigate the microvasculature of the placenta, finding no difference in macrovascular volume between normal and FGR placenta, despite a reduction in placental size [15].

Recently, we optimised a technique for placental perfusion and Micro-CT imaging without corrosion, followed by histological analysis of perfused tissue [16], which has the advantage of providing both multiscale Micro-CT and traditional histology in the same placenta. Multiscale imaging allows the whole placenta to be imaged at lower magnification, to get an overview of the vascular structure, and then blocks can be imaged at higher magnification to visualise the vascular tree down to, although not including, the terminal villi. This approach also has the benefit of allowing assessment of vascular fill with the perfusion medium.

In this study, we apply this novel imaging method to investigate the degree of heterogeneity of vascularisation within the placenta.

## 2 Method

### 2.1 Tissue preparation

#### 2.1.1 Placental perfusion

Experimental procedures were approved by Bloomsbury National Research Ethics Service Committee (REC Reference number 133888). Women undergoing elective term caesarean section following uncomplicated pregnancy at University College Hospital NHS Foundation Trust gave written consent. Placentas were taken directly from labour ward to the laboratory. In-depth discussion and justification of the perfusion process has previously been published [16]. In short, an umbilical artery was cannulated using a 22-gauge cannula, flushed with 0.9% sodium chloride with 5IU heparin/ml and sutured in place. An exit vent (approx. 1 mm) was created in the umbilical vein, and the umbilical cord was clamped distally.

The placenta was perfused with 0.9% sodium chloride with 5IU heparin/ml, using gentle manual pressure, until the fluid exiting from the vent in the umbilical vein became pink and free from blood clots. 20 ml Microfil (Flow Tech, Carver, MA), a lead based contrast agent developed for microcirculation perfusion, was then perfused through the umbilical artery cannula using gentle manual pressure until all chorionic arteries were filled, and Microfil could be seen in some of the chorionic veins. The umbilical cord was then clamped proximal to the point of cannulation, and the placenta was left at room temperature for 90 min to allow Microfil to set, as per manufacturer instructions.

A high-resolution photograph was then taken of the chorionic surface of the placenta next to a paper tape measure for scale, using a digital low-distortion single-lens reflex camera. The placenta was then placed flat in 500–750 ml 4% formalin for 48 h to fix.

#### 2.1.2 Micro-CT image acquisition

The placenta was removed from formalin, wiped dry, and placed in a vacuum sealer roll (Andrew James Vacuum Sealer Rolls) and vacuum sealed. The placenta was then mounted in a custom-made foam block and placed upright on the stage in the micro-CT scanner (XTH225 ST Micro-CT, Nikon Metrology, Tring, UK). The placenta was imaged with a Molybdenum target at 80 kV energy, 88 μA current, 1000 m s exposure time, one frame per projection, 3141 projections over 360-degree rotation, with an isotropic voxel size of 116.5 μm. The imaging time was 53 min 6 s.

The placenta was then cut into 2 cm strips as per standard histological technique. Areas of placenta that appeared well perfused were identified and full thickness blocks of 1.5–2 cm by 1.5–2 cm were taken. The location from which blocks were taken was recorded using a digital photograph. Eight blocks were taken from each placenta. Each was wrapped in parafilm and mounted in a custom-made acrylic tube, resting on a plastic stand, and imaged using a Molybdenum target, 50 kV energy, 199 μA current, 1 frame per projection, 1000 m s exposure time, 3141 projections over 360-degree rotation, with an isotropic voxel size of 13.5 μm. Each block took 53 min and 6 s to image.

**Fig. 1:**
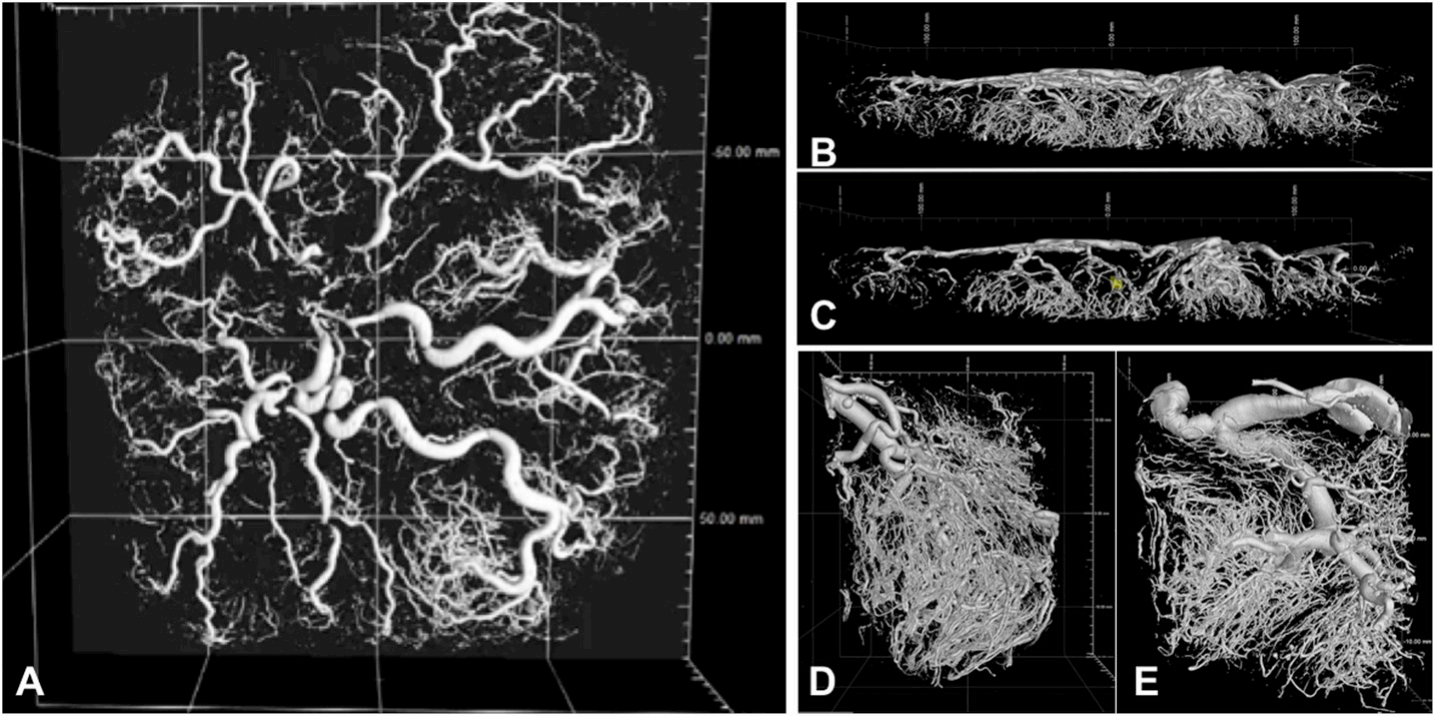
Micro-CT imaging of a human placenta perfused with Microfil. Surface renderings made using VG StudioMAX 2.2 (Volume Graphics, Germany) were thresholded halfway between the grey scale intensities of tissue and Microfil. A and B; the whole placenta, imaged with an isotropic voxel size of 116.5 μm. C; a slice through the whole placenta, showing the geometric arrangement of chorionic and villous vessels. D and E; two blocks imaged with an isotropic voxel size of 13.5 μm. The complex vascular tree is clearly seen, with whole imaging showing chorionic and stem vessels, and block imaging showing the villous vascular tree down to the terminal capillaries.

The blocks were then placed in 30 ml 4% formalin in preparation for histological analysis. The image volumes were reconstructed using a modified Feldkamp filtered back projection algorithm with proprietary software (CTPro3D; Nikon Meterology). Surface renderings of the volumes were then examined in VG Studio MAX 2.2 (Volume Graphics, Germany) to check imaging quality (Fig. 1).

#### 2.1.3 Histological slide preparation

Two 10 μm full thickness sections were taken from each block and stained with haematoxylin and eosin (H&E). For each slide, 6 micrographs at ×100 magnification were taken, three in the upper half of the tissue, close to the chorionic plate, and three in the lower half of the tissue, close to the basal plate.

### 2.2 Image analysis

#### 2.2.1 Describing tissue location in relation to cord insertion and placental edge

The high-resolution photograph of each placental chorionic plate surface was loaded into FIJI (ImageJ Version 2.0.0-rc-54/1.51f [17]), and the scale was set. The distance from the cord insertion to the centre of the site from which each block was taken, and the distance from cord insertion to placental edge through the site from which the block was taken were measured. The normalised location of the block was defined as the first distance divided by the second, multiplied by 100.

#### 2.2.2 Analysis of whole placental Micro-CT imaging

All whole placenta imaging analysis was performed in MATLAB (R2016b, MathWorks, 2016) using custom-designed algorithms. In order to analyse the data within MATLAB the whole placenta volume data was saved as a stack of TIFF files (266–492 files of 1682–2155 by 1475–2001 pixels in size). The placenta was always orientated within the stack so that each TIFF image sliced through the placenta parallel to the chorionic plate, and the distance from chorionic to basal plate increased through the stack of TIFF files.

Reading the whole placenta dataset at once was computationally prohibitive. In order to make analysis feasible on any computer, the volumes were divided into 100 (10 x 10) three-dimensional cubes, allowing smaller chunks of data to be processed. The cubes were labelled with their position in the volume and could then be re-combined.

In order to perform analysis that was relevant to placental structure, the axis of the placenta was defined. A graphical user interface was created which allowed the user to open a two-dimensional maximum intensity projection of the whole placenta stack and manually set the point of cord insertion. To define the placental edge, placenta masks were drawn. To allow analysis by distance from cord insertion, distance maps were created. The pixel distance from cord insertion to placenta edge was measured for each placenta through 360°, and then normalised from 0 to 100.

The greyscale threshold for placental tissue and Microfil filled vessels were then defined for every placenta data set. This was done in FIJI by determining the mid-point between the greyscale peaks for air and placenta as the threshold for placenta, and the point midway between the greyscale peaks for tissue and Microfil as the threshold for Microfil. This threshold was then used in MATLAB to segment the placental tissue and vascular tree of each placenta.

Once the vessels had been segmented, a vascular skeleton was created. Vessels were eroded from both sides in an iterative manner until only the centreline remained, this centreline was defined as the vascular tree skeleton. After skeletonisation, the radius of the vessel for every voxel along the skeleton was measured, as the distance from the skeletonised midline of every vessel to the boundary of the thresholded vessel.

Once vessel radius was known, vessels with a radius larger than 6-voxels (equivalent to approximately 700 μm) were excluded from further analysis, as they were though to mostly represent chorionic, not villous, vessels.

#### 2.2.3 Analysis of placental block Micro-CT imaging

To calculate the villous vascular density of each block of placental tissue, the reconstructed block volume was loaded into VG StudioMAX 2.2 (Volume Graphics, Germany). An area of interest was drawn over the bottom third of the tissue (the location for the villous vascular tree). Volumes were thresholded using the grey-scale histogram, with the threshold set at a point midway between the intensity peaks for air and tissue to segment the placenta and contrast filled vessels, and halfway between the intensity peaks for tissue and Microfil to segment the vessels perfused with Microfil. The volume of the placental tissue and of Microfil was then measured automatically, and the vascular density calculated as the volume of vessel divided by the volume of placental tissue and vessel, presented as a percentage.

#### 2.2.4 Histological analysis

**Fig. 2:**
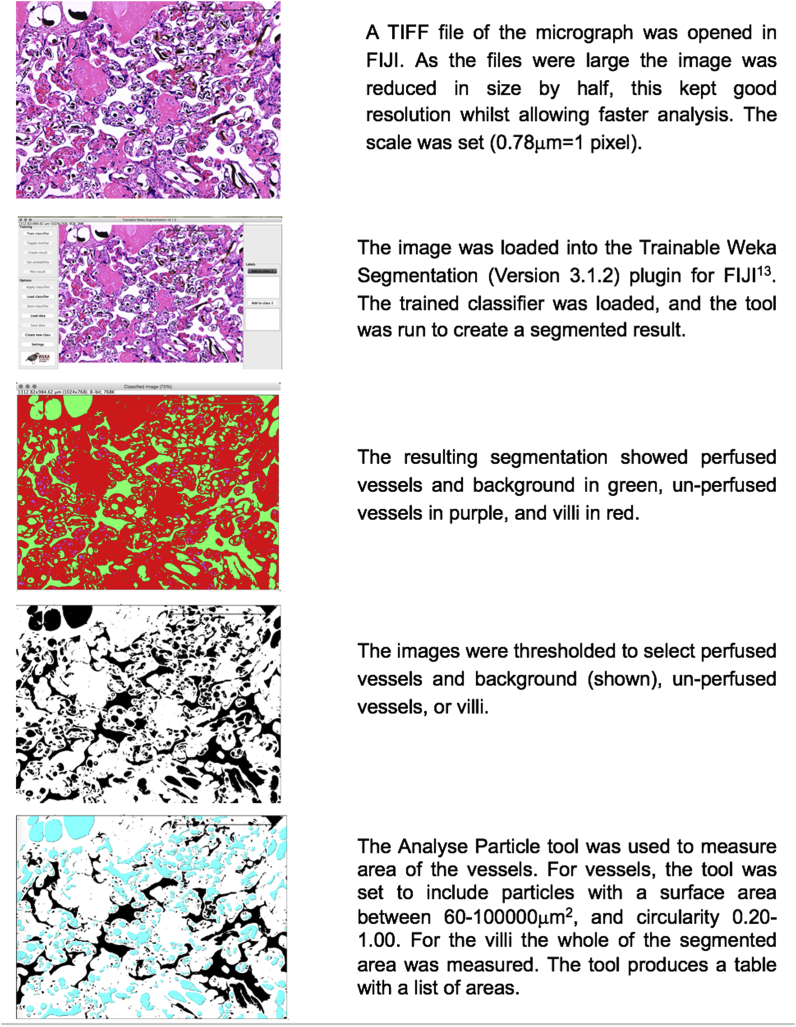
FIJI histological analysis pipeline.

A validated [18], automated pipeline, created in FIJI (ImageJ Version 2.0.0-rc-54/1.51f [17]) was used to analyse the histological sections as shown in Fig. 2. The Trainable Weka Segmentation plugin (Version 3.1.2) [19] was used to segment image features on the micrographs into three classes; perfused vessels and background (Microfil and white space (Microfil shrinks during histological processing so does not fill the whole lumen [10])), un-perfused vessels (vessels containing red cells) and villous tissue (Fig. 2).

The output images were thresholded to select the three classes defined above. The “Analyse Particle” tool was used to measure the cross-sectional area of the perfused and unperfused vessel lumens. This applies restrictions in terms of the minimum and maximal area of the particle and the circularity, and outputs a list of the area measurements for each particle within the limits. The tool was set to include particles with an area between 60–10,00,000 μm^2^ and circularity 0.20–1.00, to exclude non-vessels incorrectly segmented, and the background. For the villi, the whole of the segmented cross-sectional area was measured (Fig. 2).

The automated system output CSV files listing the perfused and un-perfused vessel lumen area and the villi area. These were input into a database, with one spreadsheet for each placenta (Microsoft Excel for Mac, Version 15.29, 2016). The vascular fill and vascular density were then calculated for each block.

Finally, a manual check was performed, by comparing each micrograph against the calculated vascular fill and density. This was to guard against limitations within the automated analysis pathway causing erroneous results.

#### 2.2.5 Statistical analysis

Data is presented as mean ± SD. Statistical analysis was performed in SPSS Statistics (IBM version 23) and MATLAB. Group comparison was done using the Kruskal-Wallis H test, with post-hoc pairwise comparison of statistically significant results using Dunn's procedure with a Bonferroni correction for multiple comparisons. Correlation was done using Spearman Rank Correlation as the test for normality was not fulfilled. Statistical significance was set at 95%.

## 3 Results

Ten placentas delivered by elective caesarean section after 38 weeks’ gestational age from uncomplicated pregnancies, with neonatal birth weight greater than the tenth centile (UK–WHO Growth Charts), were investigated (see Table 1). All women included in the study were non-smokers and did not take recreational drugs; all had an epidural for their Caesarean section (CS) birth which was a primary elective CS in 4 women and a repeat elective CS in 6 women; they did not receive any antibiotics, magnesium sulphate or oxygen resuscitation and their blood pressures remained <140/90 throughout delivery.

**Table 1:** Table showing the characteristics of the pregnancies and deliveries of the placentas included in this work.

Parameter	Clinical Characteristics of Pregnancies for Placentas Studied			
Gravidity	Median = 3	25–75% = 2-3	Range 1-5	
Parity	Median = 1	25–75% = 1-2	Range 0-3	
Gestational age (weeks)	Average = 39	SD = 0.37	Range 38 + 1–39 + 4	
Maternal age (years)	Average = 36	SD = 4.6	Range 31–46	
Ethnicity	Black = 1	White = 4	Other = 3	Unknown = 2
Birth weight (grams)	Average = 3565	SD = 414	Range 2730–4000	
Placental weight (grams)	Average = 673	SD = 58	Range 551–745	
Baby's gender	Female = 3	Male = 7		

### 3.1 Vascular fill from histology

Vascular fill was assessed as perfusion may not fill every vessel evenly with contrast agent, and unfilled vessels will not be visualised in imaging. To ensure results are representative of vascularity, not perfusion, it is essential to ensure vessels are filled, and exclude inadequately perfused tissue.

Placentas were imaged at two different resolutions, visualising different sized vessels. In order to investigate the vascular fill relevant to each resolution, we calculated it in vessels with a cross-sectional area >10,000 μm^2^ which was relevant to whole placental imaging, (n = 960 micrographs; 6 micrographs/slide, 2 slides/block, 8 blocks/placenta), and then in vessels with an area >200 μm^2^ which was relevant to block placental imaging (n = 480 micrographs; 3 micrographs/slide, 2 slides/block, 8 blocks/placenta); only the micrographs of tissue close to the basal plate were used for the analysis of vessels with an area >200 μm^2^ as this was the area analysed in imaging. The results are shown in Table 2.

**Table 2:** Table showing the vascular fill for vessels with an area >10,000 μm^2^ (vessels are within the visual resolution of whole placenta Micro-CT) and vessels with an area >200 μm^2^ (vessels within the visual resolution of the placental block Micro-CT).

Placenta	Vessels with an area >10,000 μm^2^			Vessels with an area >200 μm^2^		
	Number of blocks with vascular fill 100%	Mean Vascular Fill over all blocks (%(±SD))	Minimum Block Vascular Fill (%)	Number of blocks with vascular fill >75%	Mean Villous Vascular Fill over all blocks (% (±SD))	Minimum Block Villous Vascular Fill (%)
1	8	100 (0)	100	6	85 (21)	34
2	6	97 (8)	77	3	59 (30)	18
3	7	99 (3)	91	3	68 (26)	23
4	4	77 (40)	10	3	58 (34)	8
5	6	87 (31)	17	2	55 (20)	18
6	8	100 (0)	100	6	85 (18)	43
7	6	98 (5)	7	3	62 (23)	47
8	6	98 (3)	91	4	70 (25)	16
9	7	99 (2)	95	4	72 (19)	46
10	7	100 (1)	97	4	74 (21)	37
	N = 65	95 (14)	17	N = 38	69 (11)	8

The fill of vessels greater than 10,000 μm^2^ was generally very good, with 65 of 80 blocks having 100% fill. The lowest mean vascular fill was 77% for placenta 4. No placentas were excluded from further analysis due to poor fill. The fill of vessels with an area greater than 200 μm^2^ was less good, with 42 out of 80 blocks having vascular fill <75%. To ensure that vascular density calculations reflected vascular density rather than vascular fill, these blocks were excluded from further analysis, leaving 38 blocks, spread between the ten placentas. There was no statistically significant correlation between block location and vascular fill (r_s_ −0.009, p = 0.9), suggesting fill was not worse in peripheral compared to central placental tissue.

### 3.2 Vascular density with normalised distance from cord insertion

#### 3.2.1 Whole placental imaging

**Fig. 3:**
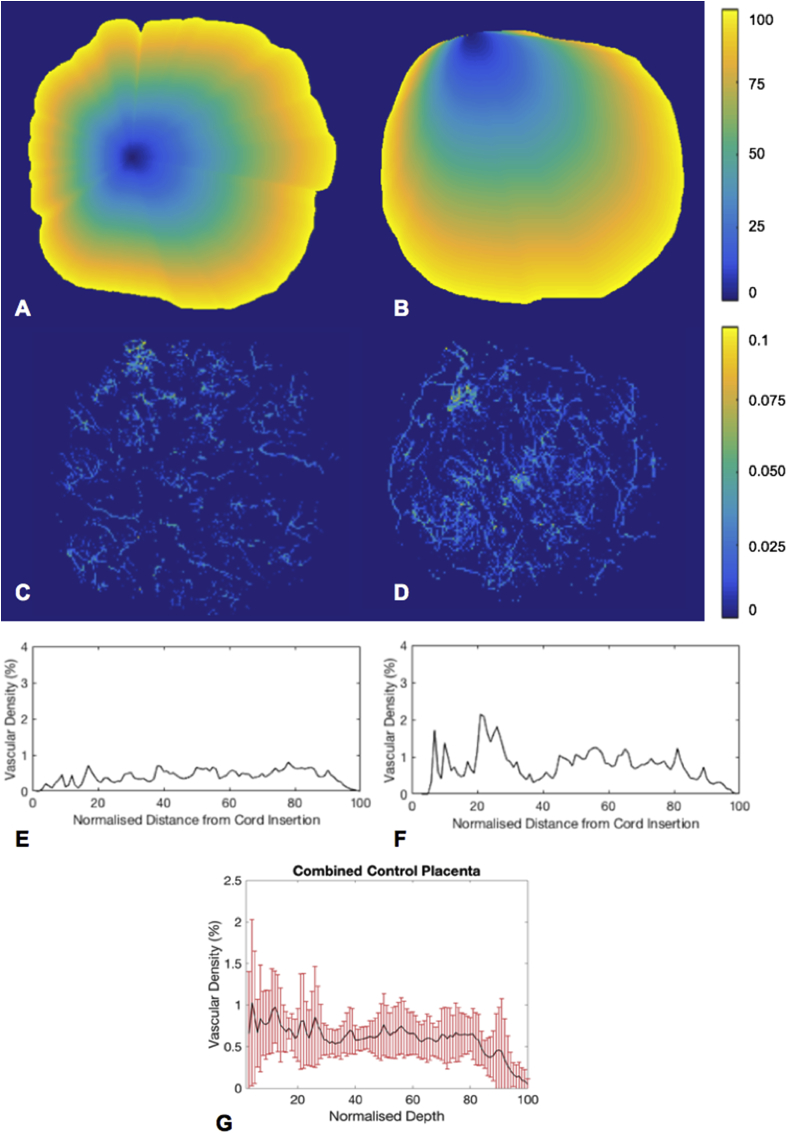
A and B; example of normalised distance maps radiating out from the umbilical cord insertion for placenta 3 (A) and 9 (B), C and D; example vascular density maps for the same placenta, E and F; graphs showing mean vascular density for each of the 100 regions from the umbilical cord insertion (0) to the placental edge (100), for each placenta. G; the combined mean vascular density with distance from the umbilical cord insertion with error bars showing standard deviation.

At the magnification achievable with Micro-CT for whole placental imaging, mean vascular density for the 10 placentas was 0.5% (SD ± 0.5, range 0.3%–1%). To investigate the relationship between villous vessel density with the distance from the umbilical cord insertion, vascular density maps were drawn for each placenta. This showed how the villous vascular density varied throughout the placental volume (Fig. 3). Using the normalised placenta distance maps the mean vascular density for each of the 100 regions from the site of the umbilical cord insertion to the placental edge was calculated and plotted (Fig. 3). There was no spatially consistent difference in villous vessel density within the main placental tissue, although there was a great degree of heterogeneity, as shown by the large error bars on the combined graph (Fig. 3). However, there was a tendency towards reduced vascular density in the peripheral 20% of the placenta, as shown by the downward trend of the combined mean (Fig. 3).

#### 3.2.2 Block placental imaging

**Fig. 4:**
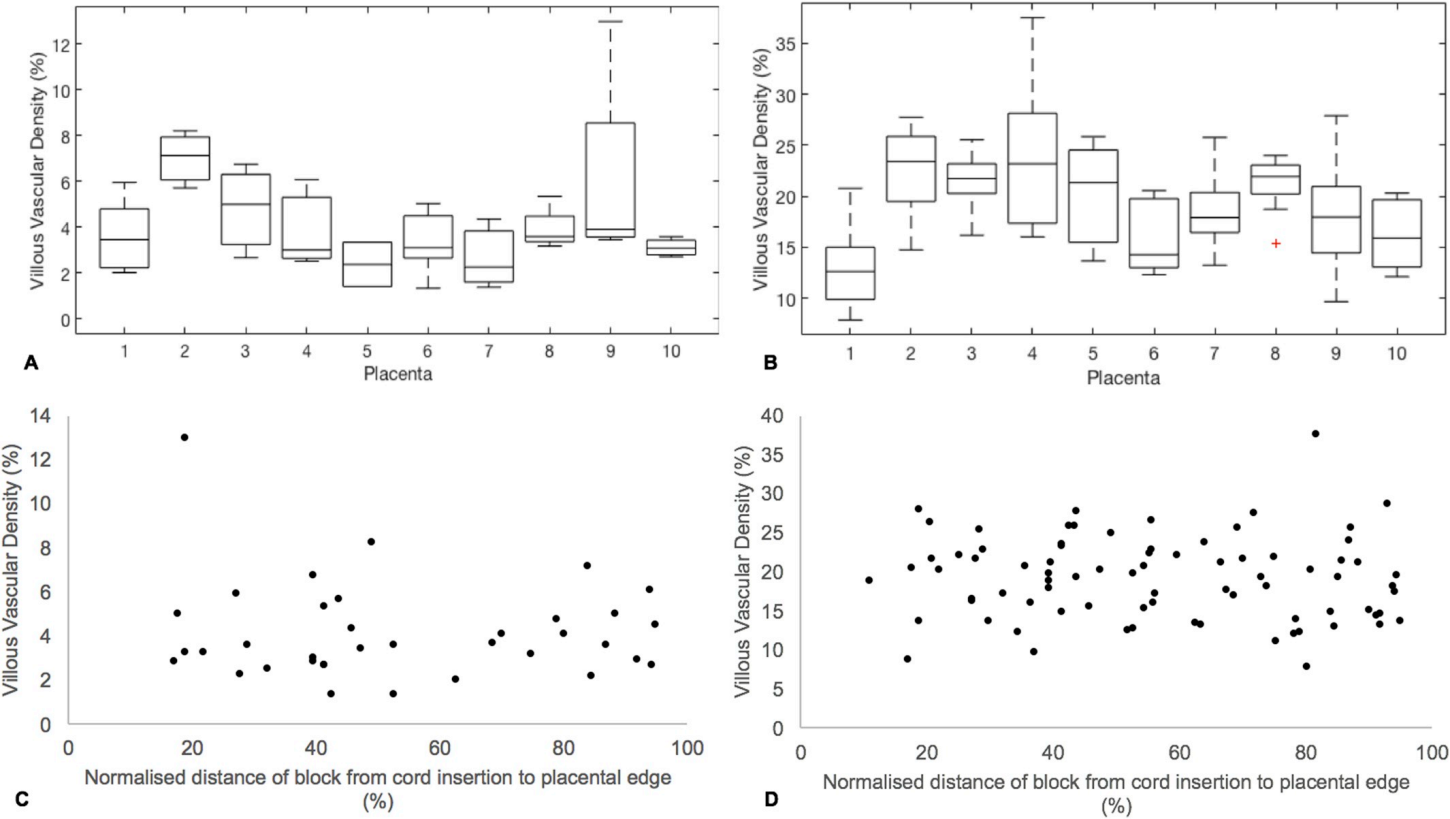
A and B: Box plots showing the spread of block vascular density (box shows 25th to 75th centile, with midline showing the median) between placentas, measured with block μCT (A) and histological analysis (B). C and D; Graphs showing correlation between block villous vascular density and normalised block location in relation to the umbilical cord insertion (0) and placental edge (100) measured with block μCT (C) and histological analysis (D).

At the magnification used for block imaging, mean vascular density for the 38 included blocks was 4% (SD ± 2%, range 1–13%). To investigate the variation in villous vascular density between placentas, a box plot was drawn (Fig. 4). Villous vascular density within one placenta commonly varied by up to 4%, representing a 100–150% increase in vascular density between blocks. There was no significant difference in the mean ranks of villous vascular density between placentas (χ^2^ = 13.06, p = 0.2). To investigate if there was a difference in villous vascular density at this resolution with distance from the umbilical cord insertion, villous vascular density was plotted against location from the umbilical cord insertion to the placental edge for each included block (Fig. 4). There was no correlation between villous vascular density and block location (r_s_ = 0.066, p = 0.7).

#### 3.2.3 Histology

The mean villous vascular density measured with histological analysis over all 80 blocks was 19% (SD ± 5%, range 8–38%). A boxplot was drawn to visualise the difference in vascular density between the placentas (Fig. 4). The vascular density of blocks from one placenta often varied by 10%. There was a significant difference in the mean ranks of villous vascular density between placentas, (χ^2^ = 30.35, p < 0.01). Post-hoc pairwise comparison showed that the significant difference in vascular density were between placenta 1 (median vascular density 12.6, IQR = 3.8%) and placentas 2 (23.4, IQR = 5.6%, p = 0.01), 3 (21.7, IQR = 2.3%, p = 0.03), 4 (23.2, IQR = 10.4%, p = 0.02) and 8 (21.9, IQR = 1.9%, p = 0.03). To investigate if there was a difference in villous vascular density on histological analysis with distance from the umbilical cord insertion, villous vascular density was plotted against location from the umbilical cord insertion to placental edge for each block (Fig. 4). There was no correlation between villous vascular density and block location (r_s_ = 0.06, p = 0.6).

## 4 Discussion

This work investigated villous vascular density in normal, term human placenta, over three scales, in relation to placental tissue location.

Whole placental micro-CT imaging was performed with an isotropic voxel size of 116.5  μm. The advantage of whole placental imaging is that it captures data throughout the placental volume, so that spatial analysis is possible. The disadvantage is that the large field of view is at the cost of magnification, so only the larger villous vessels are visible. The mean vascular density at this magnification was 0.5% (SD ± 0.51, range 0.3%–1%). No consistent spatial pattern in vascular density through the placental tissue was observed, however there was a tendency towards reduced vascular density in the peripheral 20% of the tissue.

Block placental imaging benefits from higher magnification compared to whole placental imaging, at the cost of field of view. The increased magnification allowed visualisation of vessels in the villous tree, excluding only the terminal capillaries. The mean villous vascular density at this magnification (voxel size of 13.5  μm) was 4% (SD ± 2%, range 1–13%). This shows a large degree of variability in vascular density within and between normal term placentas. When vascular density was examined in relation to tissue location between the umbilical cord insertion and placental edge, no strong correlation was found (r_s_ = 0.066, p = 0.7, powered to detect a correlation coefficient of 0.5 or greater).

Histological analysis was performed to allow visualisation of all villous vessels within the villous vascular tree, including terminal capillaries. The disadvantage of this method was that the vessels were only seen in two-dimensional cross section. Histological analysis of the villous vascular tree showed a mean vascular density of 19% (SD ± 5%, range 8–38%), consistent with previous measures in the literature [8,20]. Again, there was no strong correlation between vascular density and tissue location with respect to the distance from the umbilical cord insertion to the placental edge (r_s_ = 0.06, p = 0.6, powered to detect a correlation coefficient of 0.5 or greater).

Variation in feto-placental vascular density has been hypothesised to correspond to maternal perfusion, with evidence that the fetoplacental blood flow can be modulated to match maternal perfusion and therefore oxygenation [[21], [22], [23]]. This would facilitate efficient exchange regardless of physiological changes in maternal blood supply, that could occur daily secondary to maternal position. The mechanism by which vasoconstriction may occur is not known, with proposed mechanisms including nitric oxide released by the villous vascular tree causing vasodilation in stem arteries supplying well oxygenated areas [23], or inhibition of potassium channels causing vasoconstriction in the smooth muscle or small arterial walls in poorly oxygenated areas [24]. These theories may explain the large degree of heterogeneity in vascular density seen in this work. It is possible that more vascular dense areas represent areas of higher maternal perfusion and oxygenation in utero. In vivo techniques, such as oxygen sensitive MRI [25,26], may help us correlate in vivo perfusion and ex vivo vascular density in the future.

Our study is limited by a few issues. Ideal imaging would be capable of capturing the entire three-dimensional structure of the placental vascular tree down to the level of the terminal villi. However no imaging technology currently exists capable of both the field of view and magnification that this requires. We attempted to overcome this limitation by imaging at different magnifications but with reduced sampling volumes, and used this imaging data to investigate repeating spatial patterns in vascular density. As with all perfusion work, accuracy of results relies on good vascular fill [27]. Attempts were made to limit the effect of poor perfusion by using an optimised perfusion technique [16], by choosing well perfused tissue to image at higher resolution, and by examining tissues histologically and excluding poorly perfused tissue. By choosing well perfused tissue to image at higher magnification however, block sampling was therefore not random. This is a limitation of the work, as applying the findings of statistical analysis globally to tissue relies on the assumption that tissues were randomly sampled. To mitigate the impact of this sampling technique we ensured that the samples were taken from the whole placenta, from umbilical cord to placental edge in every case.

In this work vessels were separated from tissue using simple greyscale thresholding, and a size threshold applied to select only the villous (not chorionic) vessels. More advanced algorithms exist that may improve the segmentation, combining grey-scale thresholding and algorithms that grow the vascular tree based on proximity and similarity of grey-scale values and local vesselness properties [[28], [29], [30], [31]]. This approach would optimise the number of voxels correctly identified as vessel and minimise the noise. At present the data produced is too large and complex for available software to analyse, so further technical work is needed to optimise the vascular tree segmentation.

The main advantage of micro-CT imaging is that it captures the three-dimensional structure of the vascular tree. Improved segmentation of vessels would allow more advanced, derived analyses such as skeletonisation, which has been used by Rennie et al. to examine in detail the branching structure and tortuosity in mouse placenta [10]. This has been attempted in corrosion cast imaging with micro-CT by Junaid et al. [13]. The software they used however was limited as it was not optimised for placental data and was not capable of locating the vascular tree spatially within the placenta. This makes the branching pattern difficult to understand or analyse in a meaningful way. Data obtained using the methodology described above could be used in the development of algorithms capable of analysing features of the vascular tree such as vessel width, tortuosity and branching structure in relation to placental features such as umbilical cord insertion, chorionic vessels and placental edge. This would be an exciting application, and an important step in understanding the human placental vascular tree and how it varies in health and important obstetric pathologies, such as fetal growth restriction and pre-eclampsia.

This work presents a novel method for imaging the human placenta vascular tree using multiscale Micro-CT imaging. It demonstrates that there is a large degree of variation in vascular density throughout normal term human placentas, but does not find a reproducible spatial pattern of vascularisation between placentas. The three-dimensional data created by this technique could be used with more advanced computer analysis, to further investigate the three dimensional spatial structure of the vascular tree, and so improve our understanding of variation in normality and disease.

## Declaration of competing interest

None.
